# Female sex, high soluble CD163, and low HDL-cholesterol were associated with high galectin-3 binding protein in type 1 diabetes

**DOI:** 10.1186/s13293-019-0268-0

**Published:** 2019-11-21

**Authors:** Eva Olga Melin, Jonatan Dereke, Magnus Hillman

**Affiliations:** 1 Diabetes Research Laboratory, Lund University, Lund, Sweden; 2 Department of Research and Development, Region Kronoberg, Box 1223, SE-351 12 Växjö, Sweden

**Keywords:** Biomarkers, Depression, Galectin-3, Galectin-3 binding protein, HDL-cholesterol, Inflammation, Sex differences, sCD163, Type 1 diabetes

## Abstract

**Background:**

Galectin-3 binding protein (Gal3BP), sCD163, galectin-3, and depression have been linked to cardiovascular disease and mortality. In patients with type 1 diabetes, female sex has also been linked to cardiovascular disease and mortality. The aim was to explore whether female sex, sCD163, galectin-3, and depression were associated with Gal3BP in patients with type 1 diabetes. We adjusted for metabolic variables, creatinine, smoking, physical inactivity, and cardiovascular disease.

**Methods:**

Cross-sectional design. Patients with type 1 diabetes (*n* = 285, women 44%, age18–59 years, diabetes duration 1–55 years) were consecutively recruited from one diabetes outpatient clinic. Blood samples, anthropometrics, and blood pressure were collected, supplemented with data from electronic medical records. High Gal3BP was defined as ≥3.3 mg/l (≥80th percentile). Depression was assessed by a self-report instrument. Linear and logistic regression models were elaborated for the associations and calibrated and validated for goodness of fit with the data variables.

**Results:**

Median (q_1_, q_3_) Gal3BP was 2.3 (1.8, 3.1) mg/l. The prevalence of high Gal3BP for women was 30% and 14% for men (*p* = 0.001). Female sex (adjusted odds ratio (AOR) 3.0), sCD163 (per μg/l) (AOR 6.6), and total cholesterol (per mmol/l) (AOR 1.6) were positively associated with high Gal3BP, and HDL-cholesterol (per mmol/l) (AOR 0.2) was negatively associated with high Gal3BP.

**Conclusions:**

High Gal3BP levels were associated with female sex, increasing sCD163 and total cholesterol levels, and decreasing HDL-cholesterol levels in patients with type 1 diabetes. The prevalence of high Gal3BP was more than twice as high in the women as in the men.

## Background

Type 1 diabetes (T1D) is an autoimmune disease, characterised by insulin deficiency due to pancreatic β-cell loss leading to hyperglycaemia [1]. T1D is associated with increased risk for myocardial infarction, heart failure, and ischemic stroke [2]. There are gender differences with greatly increased coronary artery calcification (CAC) in women, but not in men with T1D [3]. Women with T1D also have a significantly greater excess risk of death from cardiovascular (CV) disease across all age groups compared to men with T1D [4].

Macrophages are major components of atherosclerotic plaques, and classically activated macrophages (M1) contribute to plaque instability [5]. Galectin-3 binding protein (Gal3BP), also known as Mac-2 binding protein or 90K, is a macrophage scavenger receptor [6]. Gal3BP binds to several galectins, including galectin-3 [7]. Gal3BP is a marker of macrophage inflammation [5] and induces a number of pro-inflammatory cytokines in human macrophages [8]. Increased plasma levels of Gal3BP have been associated with several metabolic variables and with CV and all-cause mortality [8].

Galectin-3 is a beta-galactoside-binding lectin, also known as known as Mac-2 [6, 7]. Galectin-3 is involved in several inflammatory processes [9] and marks activated macrophages in failure-prone hypertrophied hearts, predicting and contributing to cardiac dysfunction [10, 11]. Galectin-3 has been associated with coronary artery disease (CAD) and CV death in high-risk patients referred for coronary angiography [12]. It has also been linked to adverse CV outcomes in type 2 diabetes (T2D) independent of traditional risk factors [13].

CD163 is a macrophage and monocyte-expressed scavenger receptor [14]. As a result of ectodomain shedding, the extracellular portion of CD163 circulates in blood as a soluble protein (sCD163) [15]. sCD163 levels increase during inflammation and macrophage activation [15]. Increased sCD163 levels have been linked to acute coronary syndrome [16]. Increased sCD163 levels have also been linked to variables included in the metabolic syndrome and to T2D [15, 17–19]. Gal3BP and sCD163 were significantly correlated with each other, and both were associated with increased atherosclerotic lesions and lower carotid distensibility in the setting of HIV and hepatitis C virus infections [5]. We previously found that serum levels of galectin-3 were linked to circulatory sCD163 in these T1D patients [20].

Depression has been linked to immunological changes [21] and is associated with increased CV and all-cause mortality [22]. We have previously found that depression in T1D patients was associated with high galectin-3 serum levels [23], inadequate glycemic control [24], high midnight salivary cortisol secretion [25], and low HDL-cholesterol levels in T1D patients [26].

Weight gain and abdominal obesity in T1D are associated with CV risk factors and atherosclerosis [27, 28]. We previously demonstrated that the prevalence of abdominal obesity was 3.6 times higher in women compared to men with T1D [28, 29].

We hypothesised that female sex, galectin-3, sCD163, and depression, previously linked to CVD and mortality, were associated with Gal3BP in a setting of patients with T1D. We adjusted for metabolic variables, creatinine, smoking, and physical inactivity.

## Methods

### Participants and study design

**Fig. 1:**
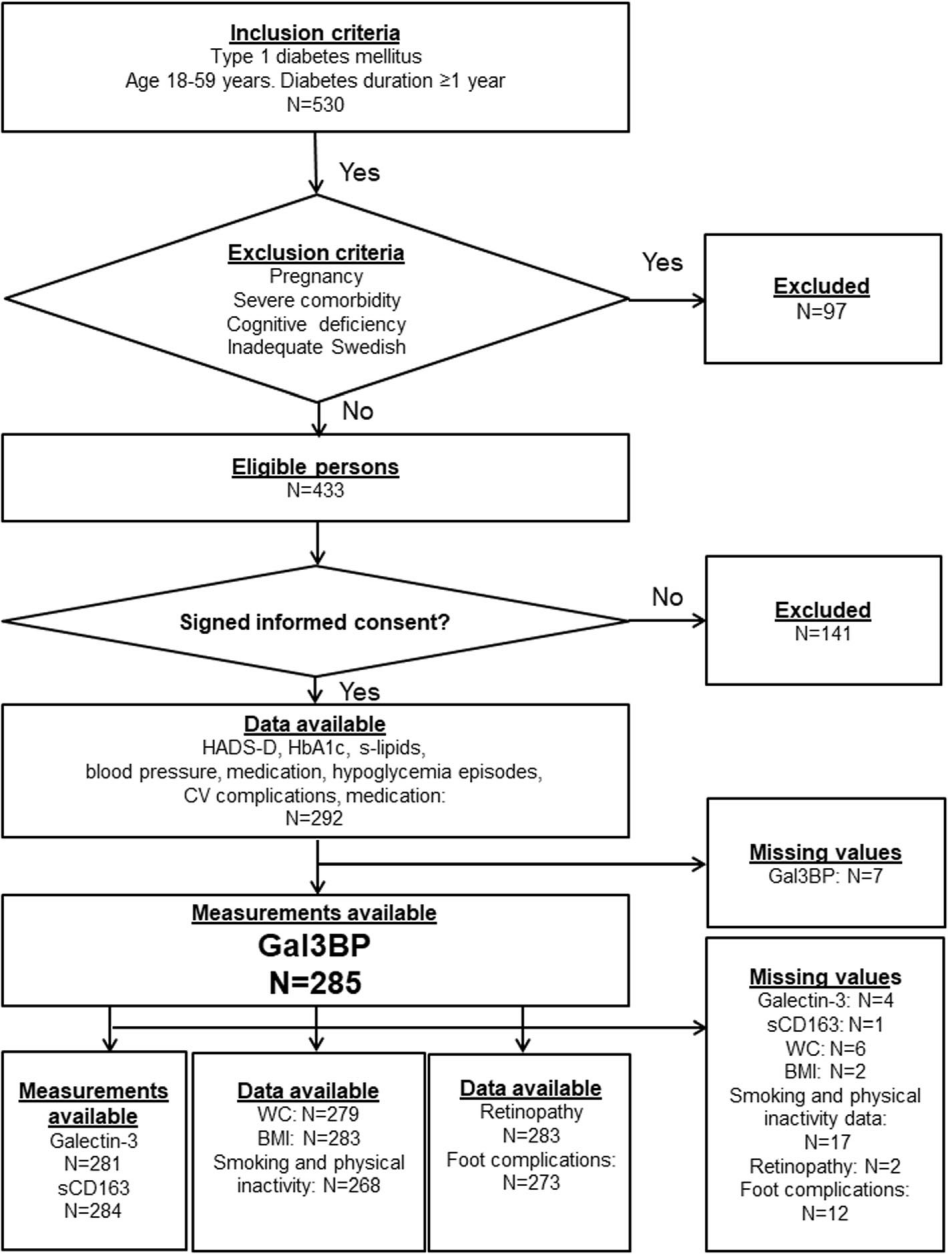
Flow chart showing inclusion and exclusion criteria and missing values

This study has a cross-sectional design and included 285 patients with T1D. Inclusion criteria were T1D with ≥1-year duration, in patients 18–59 years of age. Exclusion criteria were pregnancy; severe somatic and psychiatric disorders such as cancer, hepatic failure, and end-stage renal disease (ESRD); severe autoimmune disorders such as SLE, psychotic disorders, bipolar disorder; severe personality disorders; severe substance abuse; cognitive deficiency (due to stroke, dementia or mental retardation); and inadequate knowledge of Swedish. For inclusion and exclusion criteria, as well as missing values, see Fig. 1.The patients who attend the clinic every 6 months for regular follow-up visits were consecutively recruited by diabetes specialist physicians or diabetes specialist nurses during a 9-month period, 03/25/2009 to 12/28/2009, from one hospital diabetes outpatient clinic in Kronoberg County, Sweden. The catchment population was 125,000. Blood and saliva samples, anthropometrics, and blood pressure were collected, supplemented with data from medical records. A questionnaire was used to assess self-reported depression.

### Biochemical analyses

Plasma levels of galectin-3BP, galectin-3, and sCD163 were measured using commercially available DuoSet enzyme-linked immune-sorbent assay (ELISA) kits (R&D Systems, Minneapolis, Minnesota, USA) and optimised for human plasma [30]. The analyses were run according to the manufacturer’s instructions. The samples were diluted 1:4000, 1:2, and 1:200 for the Gal3BP, galectin-3 and sCD163 analyses, respectively. The intra-assay coefficients were 3.9%, 4.3%, and 2% for the Gal3BP, galectin-3, and sCD163 analyses, respectively. All samples were run as duplicates. Gal3BP was dichotomised at the 80th percentile.

HbA1c (mmol/mol, %) and serum lipids (mmol/l) were collected after an overnight fast and analysed with an Olympus AU clinical chemistry analyser with high specificity (Olympus AU®, Tokyo, Japan) [31]. The intra-coefficients of variation were HbA1c < 1.2%; total cholesterol  < 2.1%; HDL-cholesterol < 3.0%; LDL-cholesterol  < 2.6%; and triglycerides < 2.2%. Serum-lipids were measured directly [32].

### Anthropometrics and blood pressure

Waist circumference (WC), weight, length, and blood pressure were measured according to standard procedures by a nurse. Abdominal obesity was defined as WC ≥ 1.02 m for men and as WC ≥ 0.88 m for women. General obesity was defined as BMI ≥ 30 kg/m^2^ for both sexes [33].

### Episodes of hypoglycemia

A severe episode of hypoglycemia was defined as needing help from another person. Episodes during the last 6 months prior to recruitment were registered.

### Smoking and physical inactivity

Smokers were defined as having smoked any amount of tobacco during the last year. Physical inactivity was defined as moderate activities, such as 30 min of walking, less than once a week.

### Cardiovascular complications (CV)

CV complications were defined as ischemic heart disease, cardiac failure, stroke, or transient ischemic attack (TIA).

### Diabetic retinopathy (DR)

DR was defined as non-proliferative or proliferative retinopathy with microangiopathy changes as viewed by fundus photography through a dilated pupil.

### Foot complications

These were defined as neuropathy, angiopathy, earlier or present diabetes foot ulcer, foot infection, foot deformity, arthropathy, or amputation of the lower limb.

### Self-reported depression

Depression was defined as Hospital Anxiety and Depression Scale-Depression subscale (HADS-D) ≥8 points [34].

### Medication

Patients used either multiple daily insulin injections (MDII) or continuous subcutaneous insulin infusion (CSII).

Antidepressants (AD) were SSRIs (ATC codes N06AB04 and N06AB10); SNRIs (ATC code N06AX16); combined serotonin and norepinephrine reuptake inhibitors (ATC code N06AX21); tricyclic antidepressants (ATC code N06AA04); and/or tetracyclic antidepressants (ATC code N06AX11). The use of antidepressants was dichotomised into users and non-users of antidepressants.

Lipid lowering drugs (LLDs) were defined equal to HMG CoA-reductase inhibitors (statins), ATC-code C10AA. Indications for LLD were TC >  4.5 mmol/l (> 1.74 mg/dl) and/or LDL-cholesterol >  2.5 mmol/l (> 97 mg/dl) according to the Swedish national guidelines in 2009. The use of LLD was dichotomised into users and non-users of LLD [35].

Antihypertensive drugs (AHD) included calcium antagonists with ATC codes C08CA01-02; angiotensin-converting enzyme (ACE) inhibitors with ATC codes C09AA-BA; angiotensin II antagonists, ATC codes C09CA-DA; diuretics, ATC codes C03AA03 or C03CA01; and/or selective beta-adrenoreceptor antagonists, ATC code C07AB. Indications for AHD were systolic blood pressure > 130 mmHg and/or diastolic blood pressure >80 mmHg according to the Swedish national guidelines in 2009 [35]. The use of AHD was dichotomised into users and non-users of AHD.

### Statistical analysis

Analysis of data distribution using histograms revealed that Gal3BP, galectin-3, sCD163, and triglycerides were not normally distributed. Data were presented as median values (quartile (q)_1_, q_3_; range), and analyses were performed with Mann-Whitney *U* test. Fisher’s Exact Test (two-tailed) was used to analyse categorical data. Log-transformations were performed for Gal3BP, sCD163, and galectin-3. Linear regression analyses were performed between log-transformed galectin-3 and log-transformed Gal3BP; between log-transformed sCD163 and log-transformed Gal3BP; and between age and log-transformed Gal3BP.

Crude odds ratios (CORs) for the associations with high Gal3BP (≥3.3 mg/l) were calculated. Variables with *p* ≤ 0.10 for the CORs, and age independent of *p* value, were entered into multiple logistic regression analyses (Backward: Wald) with Gal3BP ≥3.3 mg/l as dependent variable for all, women and men. The Hosmer-Lemeshow test for goodness of fit and Nagelkerke *R*^2^ were used to evaluate each multiple logistic regression analysis model. Confidence intervals (CIs) of 95% were used. *p* < 0.05 was considered statistically significant. SPSS® version 25 (IBM, Chicago, Illinois, USA) was used.

## Results

In this study, 285 patients with T1D (56% men, age 18–59 years, diabetes duration 1–55 years) were included. The patients used either MDII (91%) or CSII (9%).

In Table 1, baseline characteristics, laboratory test results, and sex differences between the 124 women and 161 men are presented. The 124 women had higher median Gal3BP than the 161 men (*p* < 0.001).

**Table 1:** Baseline characteristics, laboratory results, and sex differences between 124 women and 161 men with T1D

Variables	All patients	Women	Men	
	*n* = 285	*n* = 124	*n* = 161	*p* value^a^
Age (years)	42 (32, 51; 18–59)	41 (30, 50()	43 (32, 52)	0.10
Diabetes duration (years)	20 (11, 30; 1–55)	19 (11, 29)	21 (11, 32)	0.27
Gal3BP ^b^ (mg/l)	2.3 (1.8, 3.1; 0.8–8.9)	2.6 (1.9, 3.6)	2.1 (1.6, 2.8)	< 0.001
sCD163 (mg/l) ^c^	0.4 (0.3, 0.5; 0.2–1.9)	0.4 (0.3, 0.6)	0.4 (0.3, 0.5)	0.78
Galectin-3 (μg/l)	0.9 (0.6–1.7; 0.001–100)	0.9 (0.5, 1.4)	0.9 (0.6, 2.0)	0.15
HbA1c				
mmol/mol	63 (54, 71; 25–110)	64 (54, 72)	63 (54, 69)	0.33
%	7.9 (7.1, 8.6; 4.4–12.2)	8.0 (7.1, 8.7)	7.9 (7.1, 8.5)	
Total cholesterol (mmol/l)	4.6 (4.1, 5.1; 2.1–10.9)	4.6 (4.1, 5.4)	4.5 (4.0, 5.1)	0.13
Triglycerides (mmol/l)	0.9 (0.7, 1.3; 0.1–5.9)	0.8 (0.6, 1.3)	0.9 (0.7, 1.3)	0.32
LDL-cholesterol (mmol/l)	2.8 (2.4, 3.3; 0.6–8.3)	2.8 (2.4, 3.4)	2.8 (2.4, 3.3)	0.79
HDL-cholesterol (mmol/l)	1.5 (1.3, 1.8; 0.3–2.7)	1.6 (1.4, 1.9)	1.4 (1.2, 1.7)	0.003
Creatinine (μmol/l) ^d^	70 (62, 182; 28–182)	62 (54, 70)	74 (68, 82)	< 0.001
Abdominal obesity ^e^	47 (17)	34 (28)	13 (8)	< 0.001^f^
General obesity ^g^	34 (12)	22 (18)	12 (8)	0.009 ^f^
Systolic BP (mmHg)	20 (110, 130; 90–160)	120 (110, 130)	125 (120, 130)	< 0.001
Diastolic BP (mmHg)	70 (70, 76; 55–100)	70 (65, 75)	70 (70, 80)	< 0.001
Smoking ^h^	27 (10)	10 (9)	17 (11)	0.55 ^f^
Physical inactivity ^i^	29 (11)	12 (10)	17 (11)	> 0.99 ^f^
Depression	30 (10)	14 (11)	16 (10)	0.85 ^f^
Cardiovascular complications	10 (4)	4 (3)	6 (4)	> 0.99 ^f^
Diabetes retinopathy ^j^	205 (72)	88 (72)	117 (73)	0.79 ^f^
Foot complications ^k^	47 (17)	13 (11)	34 (22)	0.023 ^f^
Antidepressants	23 (8)	12 (10)	11 (7)	0.39 ^f^
Antihypertensive drugs	96 (34)	35 (28)	61 (38)	0.10 ^f^
Lipid lowering drugs	133 (47)	55 (44)	78 (48)	0.55 ^f^

^a^ Mann-Whitney *U* test unless otherwise indicated. ^b^ galectin-3 binding protein. ^f^ Fisher’s Exact test. Missing values (*N*) (all/women/men): ^c^ 1/1/0; ^d^ 13/6/7; ^e^ 6/3/3; ^g^ 2/2/0; ^h, i^ 17/9/8; ^j^ 2/1/1; ^k^ 12/7/5

Log-transformed sCD163 and log-transformed Gal3BP were associated for all patients (*R*^2^ = 0.020, standardised beta coefficient 0.143, *p* = 0.016), and for men (*R*^2^ = 0.029, standardised beta coefficient 0.170, *p* = 0.031), but not for women (*R*^2^ = 0.016, standardised beta coefficient 0.128, *p* = 0.16). Log-transformed galectin-3 and log-transformed Gal3BP were not associated (all patients: *p* = 0.20; women: *p* = 0.44; men: *p* = 0.18). Age and log-transformed Gal3BP were not associated (all patients: *p* = 0.49; women: *p* = 0.51; men: *p* = 0.82).

In Table 2, comparisons are performed between patients with low and high Gal3BP for all and for each sex. High Gal3BP was defined as ≥3.3 mg/l, corresponding to the 80th percentile.

**Table 2:** Comparisons between low and high Gal3BP presented for all 285 patients and for each sex

Variables	High Gal3BP (mg/l) (≥3.3 mg/l, ≥80th percentile)								
	All patients (*n* = 285)			Women (*n* = 124)			Men (*n* = 161)		
	No	Yes	*p* value^a^	No	Yes	*p* value^a^	No	Yes	*p* value ^a^
*N*	226 (79)	59 (21)		87 (70)	37 (30)		139 (86)	22 (14)	
Gender									
Women	87 (39)	37 (63)	0.001 ^b^	–	–	–	–	–	–
Men	139 (62)	22 (37)		–	–	–	–	–	–
Age	42 (32, 51)	41 (29, 50)	0.49	42 (31, 51)	40 (30, 48)	0.63	43 (32, 51)	46 25, 55)	0.98
Diabetes duration	21 (11, 31)	18 (11, 27)	0.093	19 (10, 29)	19 (13, 28)	> 0.99	21 (13, 33)	16 (6, 26)	0.032
sCD163 (mg/l)	0.4 (0.3, 0.5)	0.5 (0.3, 0.6)	0.088	0.4 (0.3, 0.5)	0.5 (0.3, 0.6)	0.15	0.4 (0.3. 0.5)	0.5 (0.3–0.6)	0.26
Galectin-3 (μg/l)	0.9 (0.6, 1.6)	1.2 (0.7, 2.1)	0.056	0.8 (0.5, 1.4)	1.1 (0.5, 1.7)	0.24	0.9 (0.6, 1.9)	1.7 (0.8, 2.6)	0.035
HbA1c									
mmol/mol	61 (54, 69)	68 (58, 79)	0.004	63 (53, 71)	71 (59, 82)	0.013	61 (54, 68)	65 (55, 76)	0.20
%	7.8 (7.1–8.4)	8.4 (7.5–9.4)		7.9 (7.0, 8.6)	8.6 (7.5, 9.6)		7.8 (7.1, 8.4)	8.1 (7.2, 9.1)	
TC (mmol/l)	4.5 (4.1, 5.1)	4.7 (4.1, 5.3)	0.20	4.6 (4.1, 5.3)	4.7 (4.9, 5.4)	0.90	4.4 (4.0, 5.0)	4.8 (4.2, 5.1)	0.14
Triglycerides (mmol/l)	0.9 (0.6, 1.1)	1.0 (0.8, 1.6)	0.001	0.8 (0.6, 1.1)	1.0 (0.7, 1.5)	0.065	0.9 (0.7, 1.2)	1.2 (1.0, 1.8)	< 0.001
LDL-cholesterol (mmol/l)	2.8 (2.4, 3.2)	3.0 (2.4, 3.6)	0.12	2.8 (2.4, 3.3)	2.9 (2.2, 3.6)	0.82	2.8 (2.4, 3.2)	3.2 (2.6, 3.6)	0.036
HDL-cholesterol (mmol/l)	1.5 (1.3, 1.8)	1.4 (1.1, 1.7)	0.053	1.6 (1.4, 1.9)	1.5 (1.2, 1.8)	0.17	1.5 (1.2, 1.8)	1.3 (1.1, 1.5)	0.024
Creatine (μmol/l)	70 (63–80)	66 (53–76)	0.005	64 (56, 70)	61 (52, 69)	0.17	74 (68, 82)	72 (66, 80)	0.30
Abdominal obesity	37 (16)	10 (18)	0.84 ^b^	25 (29)	9 (26)	> 0.99 ^b^	12 (9)	1 (5)	> 0.99 ^b^
General obesity	20 (9)	14 (25)	0.002 ^b^	14 (16)	8 (23)	0.44 ^b^	6 (4)	6 (27)	0.002 ^b^
Systolic BP (mmHg)	120 (115, 130)	120 (110, 130)	0.37	120 (110, 130)	115 (110, 130)	0.57	125 (120, 130)	128 (110, 136)	0.69
Diastolic BP (mmHg)	70 (70,76)	70 (65, 75)	0.48	70 (65, 75)	70 (62, 70)	0.27	70 (70, 80)	75 (70, 80)	0.19
Smoking	21 (10)	6 (11)	0.80 ^b^	14 (16)	8 (23)	0.44 ^b^	14 (10)	3 (15)	0.47 ^b^
Physical inactivity	19 (9)	10 (18)	0.085 ^b^	6 (7)	6 (18)	0.18 ^b^	13 (10)	4 (19)	0.26 ^b^
Depression	21 (9)	9 (15)	0.23 ^b^	8 (9)	6 (16)	0.35 ^b^	8 (9)	6 (16)	0.35 ^b^
Cardiovascular complications	6 (3)	4 (7)	0.22 ^b^	1 (1)	3 (8)	0.079 ^b^	5 (4)	1 (4)	0.59 ^b^
Diabetes retinopathy	162 (72)	43 (73)	> 0.99 ^b^	58 (67)	30 (81)	0.14 ^b^	104 (75)	13 (59)	0.12 ^b^
Foot complications	36 (16)	11 (20)	0.55 ^b^	7 (8)	6 (18)	0.19 ^b^	29 (22)	5 (23)	> 099 ^b^
Antidepressants	16 (7)	7 (12)	0.28 ^b^	5 (6)	7 (19)	0.041 ^b^	11 (8)	0	0.36 ^b^
Antihypertensive drugs	77 (34)	19 (32)	0.88 ^b^	22 (25)	13 (35)	0.28 ^b^	55 (40)	6 (27)	0.35 ^b^
Lipid lowering drugs	103 (46)	30 (51)	0.56 ^b^	36 (41)	19 (51)	0.33 ^b^	67 (48)	11 (50)	> 0.99 ^b^

^a^ Mann-Whitney *U* test unless otherwise indicated. ^b^ Fisher’s Exact test

Abdominal obesity: women *p* >0.99^b^, men *p* >0.99^b^

General obesity: women *p*=0.44^b^, men= 0.002^b^

The prevalence of high Gal3BP was 2.1 times higher in the women than in the men (30% versus 14%) (*p* = 0.001). Comparisons between 59 patients with high Gal3BP and 226 patients with low Gal3BP showed that the patients with high Gal3BP had higher medians of HbA1c (*p* = 0.004) and triglycerides (*p* = 0.001) and higher prevalence of general obesity (*p* = 0.002). The 37 women with high Gal3BP levels compared to the 87 women with low Gal3BP levels had higher median HbA1c (*p* = 0.013) and higher prevalence of antidepressant use (*p* = 0.041). The 22 men with high Gal3BP levels compared to the 139 men with low Gal3BP levels had higher medians of galectin-3 (*p* = 0.035), triglycerides (*p* < 0.001), and LDL-cholesterol (*p* = 0.036); higher prevalence of general obesity (*p* = 0.002); and lower median HDL-cholesterol (*p* = 0.024).

In Table 3, variables associated with high Gal3BP are presented for all and for each sex. In all patients, female sex (AOR 3.0), sCD163 (per mg/l) (AOR 6.6), and total cholesterol (per mmol/l) (AOR 1.6) were positively associated, and HDL-cholesterol (per mmol/l) (AOR 0.2) was negatively associated with high Gal3BP. In the women, HbA1c (per mmol/mol) (AOR 1.03) was associated with high Gal3BP. In the men, sCD163 (per mg/l) (AOR 11.2), total cholesterol (per mmol/l) (AOR 2.3), and general obesity (AOR 7.9) were positively associated, and HDL-cholesterol (per mmol/l) (AOR 0.2) was negatively associated with high Gal3BP.

**Table 3:** Associations with high Gal3BP for all, women and men

Variables	High galectin-3BP (≥3.3 mg/l, ≥80th percentile)							
	All patients				Women		Men	
	COR	*p* value	AOR	*p* value^a^	AOR	*p* value^b^	AOR	*p* value^c^
Sex (women)	2.7 (1.5–4.9)	0.001	3.0 (1.5–5.9)	0.001	–	–	–	–
Age	0.99 (0.97–1.02)	0.43	1.02 (0.99–1.05)	0.22	0.98 (0.94–1.02)	0.27	1.03 (0.98–1.08)	0.30
Diabetes duration	0.98 (0.95–1.00)	0.076	0.98 (0.95–1.00)	0.11	–	–	0.96 (0.92–1.01)	0.14
sCD163 (mg/l)	5.4 (1.6–18.0)	0.005	6.6 (1.9–23.2)	0.003	4.6 (0.8–26.9)	0.093	11.2 (2.0–64.5))	0.007
Galectin-3 (μg/l)	1.04 (0.99–1.10)	0.14	–	–	–	–	–	–
HbA1c mmol/mol	1.03 (1.01–1.05)	0.008	1.02 (1.00–1.04)	0.09	1.03 (1.00–1.06)	0.039	–	–
TC (mmol/l)	1.4 (1.0–1.8)	0.022	1.6 (1.1–2.2)	0.007	–	–	2.3 (1.3–3.9)	0.003
Triglycerides (mmol/l)	1.5 (1.1–2.1)	0.014	0.9 (0.5–1.5)	0.58	–	–	0.7 (0.4–1.5)	0.41
LDL-cholesterol (mmol/l)	1.5 (1.1–2.2)	0.017	0.6 (0.1–3.5)	0.53	1.1 (0.6–1.8)	0.76	0.4 (0.03–6.6)	0.56
HDL-cholesterol (mmol/l)	0.45 (0.2–1.0)	0.059	0.2 (0.1–0.7)	0.010	–	–	0.2 (0.02–0.9)	0.040
Creatinine	0.99 (0.97–1.01)	0.16	–	–	–	–	–	–
Abdominal obesity	1.1 (0.5–2.4)	0.77	–	–	–	–	–	–
General obesity	3.4 (1.6–7.2)	0.002	1.6 (0.7–4.0)	0.29	–	–	7.9 (1.9–32.6)	0.004
SBP	0.99 (0.96–1.01)	0.26	–	–	–	–	–	–
DBP	1.00 (0.96–1.04)	0.83	–	–	–	–	–	–
Smoking	1.2 (0.5–3.1)	0.74	–	–	–	–	–	–
Physical inactivity	2.3 (1.0–5.2)	0.053	1.8 (0.7–4.8)	0.22	–	–	–	–
Depression	0.6 (0.2–1.3)	0.19	–	–	–	–	–	–
CV complications	2.7 (0.7–9.8)	0.14	–	–	4.0 (0.3–47.3)	0.26	–	–
Diabetes retinopathy	1.0 (0.5–2.0)	0.93	–	–	–	–	–	–
Foot complications	1.3 (0.6–2.7)	0.54	–	–	–	–	–	–
AD	1.8 (0.7–4.5)	0.24	–	–	2.6 (0.7–9.4)	0.15	–	–
AHD	0.9 (0.5–1.7)	0.79	–	–	–	–	–	–
LLD	1.2 (0.7–2.2)	0.47	–	–	–	–	–	–

^a, b, c^ Logistic regression analyses (Backward: Wald): variables with *p* values ≤0.10 for the CORs and age were included in the analyses; *N* = ^a^ 267/^b^ 123/^c^ 160; Nagelkerke *R*^2^: ^a^ 0.206/ ^b^ 0.089/^c^ 0.298; Hosmer-Lemeshow test: ^a^ 0.991/^b^ 0.142/^c^ 0.821

## Discussion

In this study of 285 patients with T1D, high Gal3BP levels (≥3.3 mg/l) were associated with female sex, increasing sCD163 and total cholesterol levels, and decreasing HDL-cholesterol levels. The prevalence of high Gal3BP was more than twice as high in the women as in the men. In the women, high Gal3BP levels were associated with HbA1c. In the men, high Gal3BP levels were associated with increasing sCD163 and total cholesterol levels, decreasing HDL-cholesterol levels, and general obesity. High Gal3BP was neither associated with galectin-3 nor depression.

The first strength of this study is that the population of patients with T1D was well-defined. Patients with severe somatic or psychiatric comorbidities and/or substance abuse were excluded, as well as pregnant women. Of particular importance are that no patients with ESRD were included as ESRD is accompanied by immune dysfunction [36] and that no patients with a severe autoimmune disorder such as SLE, liver cirrhosis, or cancer were included as Gal3BP is involved in several of these conditions [8, 37, 38]. Second, we have included relevant variables as disturbances of sCD163, galectin-3, and metabolic variables previously have been linked to CVD [4, 5, 8, 10, 11, 13, 16, 28, 32, 33, 39]. Depression, smoking, and physical inactivity were also included due to their previously demonstrated impact on CVD and mortality [22, 40, 41]. Third, precise ELISA techniques were used. The commercial ELISA assay showed low intra-assay coefficients of variation for Gal3BP, sCD163, and galectin-3.

One limitation was that the number of patients with CV complications was low, so we could neither confirm nor exclude any association between Gal3BP and CV complications. Other limitations were that we have not measured any sex hormones and there were no data available regarding menopause. However, we did not find any correlation between Gal3BP and age, so there was no indication that menopause was of particular importance for determining the Gal3BP levels.

To our knowledge, we are the first to explore the associations between Gal3BP and sex, galectin-3, sCD163, depression, metabolic factors, and life style variables in patients with T1D. We have not found any previous study exploring Gal3BP levels in a population of T1D patients. One study states that Gal3BP levels are higher in patients with diabetes, but the authors did not distinguish between T1D and T2D [8].

To include sex in the analyses is of particular importance as CAC is greatly increased in women with T1D [3] and as women compared to men with T1D are at higher risk for CV death across all age groups [4]. To stratify for sex is also of utmost importance while performing autoimmune disease biomarker research [42]. Several sex differences of macrophage function, including activation levels, phagocytic capacity, and cytokine production, have been demonstrated [42]. Numerous cytokines released by macrophages are modulated by oestradiol, progesterone, or androgens [42]. We have not found any previous study exploring sex differences and the impact of sex hormones on galectin-3BP. We have only found one study that showed that Gal3BP levels could be modulated by hormones [43]. The explored hormones were TSH, insulin, and IGF-I, which all had modulation capacity [43].

We found an association between Gal3BP and sCD163 which is in accordance with previous research in the setting of HIV and HCV infections, where these two biomarkers were correlated with each other [5]. In that study, both Gal3BP and sCD163 were associated with increased atherosclerotic lesions [5]. We did not find any association between galectin-3 and Gal3BP, which previously have been linked to each other in the context of cancer [7]. We have not found any studies exploring links between galectin-3 and Gal3BP in the context of T1D or CVD. In previous research, BMI and triglycerides were positively associated with Gal3BP, while HDL-cholesterol levels were negatively associated with Gal3BP [8]. Despite a much higher prevalence of obesity in the women than in the men, neither abdominal nor general obesity was associated with high Gal3BP in the women. General obesity was associated with high Gal3BP in the men only. The triglyceride levels were higher in patients with high Gal3BP, but there was no independent association between Gal3BP and triglycerides, which differs from previous research [8]. An association between lower HDL-cholesterol levels and high Gal3BP levels was demonstrated in the men. The finding of an association between lower HDL-cholesterol and high Gal3BP levels is interesting as HDL-cholesterol protects against atherosclerosis by removing excess cholesterol from macrophages by the reverse cholesterol transport and additionally exerts anti-inflammatory actions [44]. Systemic and vascular inflammation have been proposed to convert HDL-cholesterol to a dysfunctional form that has impaired antiatherogenic effects, and even pro-inflammatory effects with increased risk for atherosclerosis [44]. Increasing HbA1c levels were associated with high Gal3BP levels in women only. To our knowledge, the association between HbA1c and high Gal3BP has not been explored in patients with T1D previously. Smoking and physical inactivity, which are two factors contributing to CVD [40, 41], were not associated with Gal3BP in this study, and not with sCD163 and galectin-3 in our previous studies [20, 23].

In previous research, increased plasma levels of Gal3BP have been associated with CV and all-cause mortality [8]. It is not clarified whether Gal3BP is just a marker of macrophage activation and increased risk for CVD and mortality or whether Gal3BP directly contributes to CVD and mortality [8]. The effects of increased Gal3BP on CVD and mortality might be mediated by associated metabolic and inflammatory disturbances [8]. If there is a direct impact of Gal3BP on CVD and mortality, our findings of increased Gal3BP levels in women might contribute to the increased prevalence of CAC and CV death observed in women with T1D [3, 4].

In future research, the exploration of sex differences of Gal3BP in a larger population of patients with T1D is suggested. Another subject for exploration is the impact of sex hormones on Gal3BP. High Gal3BP levels indicate increased risk for CV and all-cause mortality according to previous research [8]. Comparing Gal3BP to other metabolic and inflammatory risk factors will be necessary in prospective studies, to evaluate whether Gal3BP is an independent risk factor for CV disease and mortality. We are therefore planning an 11-year follow-up of this study. Whether high Gal3BP levels can be a target for treatment is also a subject for further research.

## Conclusions

Female sex, sCD163, and total cholesterol were positively associated, and HDL-cholesterol was negatively associated with high Gal3BP in patients with T1D. The prevalence of high Gal3BP was more than twice as high in the women as in the men.

### Perspectives and significance

The increased prevalence of high Gal3BP demonstrated in the women might contribute to the increased risk for CAC and CV death previously demonstrated in women with T1D. The effects of high Gal3BP on CV disease might be direct, alternately, might be mediated by associated metabolic and inflammatory disturbances. This has to be further explored in longitudinal studies. Potentially, after further research, high Gal3BP might be used either as a valuable risk marker or as a treatment target.

## Data Availability

All data are saved at SPSS files for 15 years at the Department of Research and Development, Region Kronoberg, Växjö, Sweden. The data sets are not available publicly as individual privacy could be compromised. The data set is available from the corresponding author on reasonable request.
